# Proteomics Approaches for Biomarker and Drug Target Discovery in ALS and FTD

**DOI:** 10.3389/fnins.2019.00548

**Published:** 2019-06-11

**Authors:** Thomas J. Hedl, Rebecca San Gil, Flora Cheng, Stephanie L. Rayner, Jennilee M. Davidson, Alana De Luca, Maria D. Villalva, Heath Ecroyd, Adam K. Walker, Albert Lee

**Affiliations:** ^1^Neurodegeneration Pathobiology Laboratory, Queensland Brain Institute, The University of Queensland, St Lucia, QLD, Australia; ^2^Centre for Motor Neuron Disease Research, Department of Biomedical Sciences, Faculty of Medicine and Health Sciences, Macquarie University, North Ryde, NSW, Australia; ^3^School of Chemistry and Molecular Bioscience, University of Wollongong, Wollongong, NSW, Australia; ^4^Illawarra Health and Medical Research Institute, Wollongong, NSW, Australia

**Keywords:** amyotrophic lateral sclerosis, frontotemporal dementia, proteomics, mass spectrometry, protein aggregation

## Abstract

Neurodegenerative disorders such as amyotrophic lateral sclerosis (ALS) and frontotemporal dementia (FTD) are increasing in prevalence but lack targeted therapeutics. Although the pathological mechanisms behind these diseases remain unclear, both ALS and FTD are characterized pathologically by aberrant protein aggregation and inclusion formation within neurons, which correlates with neurodegeneration. Notably, aggregation of several key proteins, including TAR DNA binding protein of 43 kDa (TDP-43), superoxide dismutase 1 (SOD1), and tau, have been implicated in these diseases. Proteomics methods are being increasingly applied to better understand disease-related mechanisms and to identify biomarkers of disease, using model systems as well as human samples. Proteomics-based approaches offer unbiased, high-throughput, and quantitative results with numerous applications for investigating proteins of interest. Here, we review recent advances in the understanding of ALS and FTD pathophysiology obtained using proteomics approaches, and we assess technical and experimental limitations. We compare findings from various mass spectrometry (MS) approaches including quantitative proteomics methods such as stable isotope labeling by amino acids in cell culture (SILAC) and tandem mass tagging (TMT) to approaches such as label-free quantitation (LFQ) and sequential windowed acquisition of all theoretical fragment ion mass spectra (SWATH-MS) in studies of ALS and FTD. Similarly, we describe disease-related protein-protein interaction (PPI) studies using approaches including immunoprecipitation mass spectrometry (IP-MS) and proximity-dependent biotin identification (BioID) and discuss future application of new techniques including proximity-dependent ascorbic acid peroxidase labeling (APEX), and biotinylation by antibody recognition (BAR). Furthermore, we explore the use of MS to detect post-translational modifications (PTMs), such as ubiquitination and phosphorylation, of disease-relevant proteins in ALS and FTD. We also discuss upstream technologies that enable enrichment of proteins of interest, highlighting the contributions of new techniques to isolate disease-relevant protein inclusions including flow cytometric analysis of inclusions and trafficking (FloIT). These recently developed approaches, as well as related advances yet to be applied to studies of these neurodegenerative diseases, offer numerous opportunities for discovery of potential therapeutic targets and biomarkers for ALS and FTD.

## Introduction

Neurodegenerative diseases, including amyotrophic lateral sclerosis (ALS) and frontotemporal dementia (FTD), demonstrate dysfunctional protein clearance and accumulation of protein-rich inclusions in neuronal cells. Resolving whether these inclusions are a cause of cellular degeneration or a symptom of homeostatic dysfunction has proven difficult, and the pathological mechanisms underlying their formation are still largely unknown. However, through attempting to answer these questions, the fundamental roles of protein aggregation and protein clearance in the pathology of these diseases have been established. Recent advances in proteomics technologies have advanced our understanding of both the primary pathological proteins as well as other associated proteins that may play downstream roles in disease mechanisms. The purpose of this review is to evaluate the current proteomic tools and techniques being used to understand these diseases, to summarize the state of knowledge gained from proteomic studies on ALS and FTD, and to discuss development of more effective disease-modifying treatments and biomarkers for clinical assessment driven by new advances in proteomic technologies.

### Pathology and Disease Mechanisms of ALS and FTD

ALS and FTD are both debilitating neurodegenerative diseases caused by selective loss of subsets of neurons. In ALS, disease pathology primarily affects the motor neurons of the primary motor cortex and spinal cord (Foster and Salajegheh, 2019), and in FTD, neurons of the frontal and temporal lobe are the primary targets of degeneration (Olney et al., 2017). Characteristic of these neurodegenerative diseases is the accumulation of protein inclusions in the cytoplasm of neurons (Ling et al., 2013). These pathological proteins, which may be affected by inherited mutations in familial disease or undergo unclear pathological changes in sporadic cases of disease, include but are not limited to TAR DNA binding protein of 43 kDa (TDP-43), superoxide dismutase 1 (SOD1) and microtubule-associated protein tau (Ling et al., 2013). Numerous mutations in these and other proteins have been shown to individually cause either ALS or FTD, or indeed both diseases in the same patient or within the same family; however, the vast majority of ALS cases and approximately half of all FTD cases have no known underlying dominant mechanism of inheritance (Ling et al., 2013; Nguyen et al., 2018).

Prominent amongst these pathological proteins is TDP-43, which is a highly conserved predominantly nuclear protein with functional roles in RNA metabolism (Ayala et al., 2008; Weskamp and Barmada, 2018). TDP-43 has been reproducibly identified as one of the components of cytosolic protein inclusions, a pathological hallmark in almost all ALS and some FTD cases (known pathologically as frontotemporal lobar degeneration with TDP-43 pathology, FTLD-TDP), irrespective of genetic inheritance or mutation (Neumann et al., 2006, 2007; Wang et al., 2008). Aberrant cytoplasmic TDP-43 has been reported in neurons and glial cells of the central nervous system in patients with either ALS or FTD (Arai et al., 2006; Neumann et al., 2006), and TDP-43 is therefore a primary target for studies of mechanisms of these diseases. Indeed, mutations in numerous other ALS-linked genes, including *C9ORF72*, are also characterized by the presence of TDP-43 pathology (Chew et al., 2015).

Although TDP-43 is the main pathological protein in almost all ALS cases, the first known causative mutations linked to ALS were identified in the protein SOD1 (Rosen et al., 1993), and these account for approximately 20% of all familial ALS cases (Pasinelli and Brown, 2006). SOD1 is a cytosolic protein responsible for catalyzing the breakdown of harmful superoxide radicals (Field et al., 2003; Sea et al., 2015). However, disease-causative mutations in the SOD1 protein variably affect protein function, and knockout of SOD1 in animals does not result in an ALS-like disease, such that the primary mechanism of toxicity of mutant SOD1 is considered to be a gain-of-function even if alterations in SOD1 function may modify disease presentation (Valentine et al., 2005; Saccon et al., 2013). Mutant SOD1 forms large intraneuronal inclusions in people with ALS-linked SOD1 mutations as well as in cellular and animal model systems (Bruijn et al., 1998; Kato et al., 2000; Ayers et al., 2017).

Unlike SOD1, tau is a primarily neuronal axoplasmic protein that stabilizes microtubules (Kadavath et al., 2015). Numerous other roles for tau have been also been described, such as inhibition of HDAC6, a protein involved in tubulin acetylation (Perez et al., 2009), and in intraneuronal transport (Bodea et al., 2016). Importantly, phosphorylation of tau has been shown to correlate with the formation of tau inclusions that are present in tissue from people with FTD (Vega et al., 2005), and mutations within the *MAPT* gene encoding tau are a prominent cause of non-TDP-43-associated cases of FTD (Rademakers et al., 2004). Indeed, aggregation of tau and alterations in tau function are prominent in FTLD-tau as well as other neurodegenerative diseases, including Alzheimer’s disease (Frost et al., 2015).

Overall, numerous mechanisms have been implicated in the pathogenesis of these diseases, related to mutations and/or dysfunctions which impact on neuronal viability via changes in numerous pathways including intracellular transport, cellular stress responses, RNA metabolism and protein clearance machinery (Walker and Atkin, 2011; Ling et al., 2013; Zhang et al., 2015; Tank et al., 2018). However, despite the diversity of possible upstream causes of disease, the prominence of protein aggregation suggests that this plays a key role in driving neurodegeneration in ALS and FTD.

### Proteostasis and Protein Aggregation in ALS and FTD

Proteins are the functional components that drive the majority of cellular processes. Protein homeostasis or “proteostasis” describes a network of constitutively expressed housekeeping and cellular stress-inducible molecular pathways that maintain proteins in a biologically active conformation, or degrade them, to ensure that cell viability is not compromised (Balch et al., 2008; Hipp et al., 2014). The proteostasis network can be clustered into several pathways including the heat shock response, unfolded protein response, ubiquitin-proteasome system (UPS), and autophagy machinery (Webster et al., 2017). Under physiological conditions, the mechanisms of proteostasis function sufficiently to maintain cell viability. However, if proteostasis deteriorates or becomes overwhelmed, for example in the context of ALS and FTD, aberrant protein accumulation and aggregation can occur, and cell viability may be threatened.

Under normal cellular conditions, proteins exist in their native conformation, consisting of external hydrophilic surfaces and an internal hydrophobic core. Apart from the folding that occurs for nascent polypeptides as they are synthesized on the ribosome, protein folding and unfolding occurs at other important times during the lifespan of many proteins. For example, proteins unfold and are refolded during trafficking across intracellular membranes, cellular secretion, and during times of cellular stress (Kincaid and Cooper, 2007; Gregersen and Bross, 2010). When proteins are subjected to cellular stresses, such as oxidative stress or increased burden to mitochondria or the endoplasmic reticulum, they may unfold and form partially folded protein intermediates that expose the hydrophobic regions of the polypeptide to the cytosol, which are otherwise buried within the protein (Hipp et al., 2014). Exposed hydrophobic regions are attracted to similar hydrophobic regions on adjacent partially folded protein intermediates, which may aggregate together and enter thermodynamically favorable pathways that lead to the formation of higher-order oligomers (Stefani, 2008). These oligomers may be toxic and also form the building blocks of larger aggregates and protein inclusions in neurodegenerative diseases (Lasagna-Reeves et al., 2012; Blair et al., 2013; Ait-Bouziad et al., 2017; Shafiei et al., 2017).

The maintenance of functional proteostasis to ameliorate protein aggregation is particularly important in post-mitotic cells such as neurons, since disrupted proteostasis cannot be simply counteracted by apoptosis and replacement with new healthy neurons, unlike most other cell types (Morimoto, 2008). A recent review has discussed evidence that cellular stress in the spinal cord of the SOD1^G93A^ mouse, the most widely used model of ALS, does not result in the induction of the anti-aggregation heat shock response, which may suggest that this pathway is impaired in disease (San Gil et al., 2017). Impairment of proteasomal degradation likely also contributes to the accumulation of ubiquitinated proteins, including SOD1 and TDP-43 (Cheroni et al., 2005, 2009; Scotter et al., 2014). Numerous factors, such as activation of cellular stress responses and dysfunctions in proteasome activity and autophagy, may contribute to varying degrees to the accumulation of proteins in disease, although the underlying mechanisms of inclusion formation and associated pathophysiology remain unclear (Tanaka and Matsuda, 2014). However, since protein aggregation and inclusion formation are hallmarks of these diseases, understanding these processes, and how protein aggregation perturbs neuronal function, is likely to reveal new ways to delay or prevent neurodegeneration.

Proteins that are supersaturated, meaning that their concentration in the cell is greater than their predicted solubility, also have a higher propensity to aggregate (Ciryam et al., 2017). In ALS and FTD, proteins such as TDP-43 have been shown to be supersaturated due to their unusually high concentration (Ciryam et al., 2017). Phase transitions may result in proteins precipitating out in the cytoplasm, a liquid-liquid separation, and proteins with low complexity domains such as TDP-43 may be more prone to this transition (Li et al., 2018). Indeed, recently RNA has been proposed as a regulator of protein phase separation, suggesting that dysregulation of RNA: protein interactions may contribute to the formation of protein aggregates (Maharana et al., 2018; Zacco et al., 2019).

Analyzing how disease-related proteins aggregate, identification of the interaction partners of native and aggregated pathological proteins, and determination of the consequences of protein aggregation within neurons will likely elucidate the patho-mechanisms involved in ALS and FTD. The remainder of this review will focus on the methods and outcomes of previous and potential future studies using proteomics technologies to address these fundamental issues. By developing an understanding of the numerous tools available for these experiments and devising the most relevant biological questions, these studies offer the potential to elucidate the mechanisms of pathogenesis of ALS/FTD, and to identify biomarkers for these diseases. Together, this information may allow the future development of better treatments for people living with ALS and FTD.

## Proteomic Approaches to Study Als and Ftd

**FIGURE 1:**
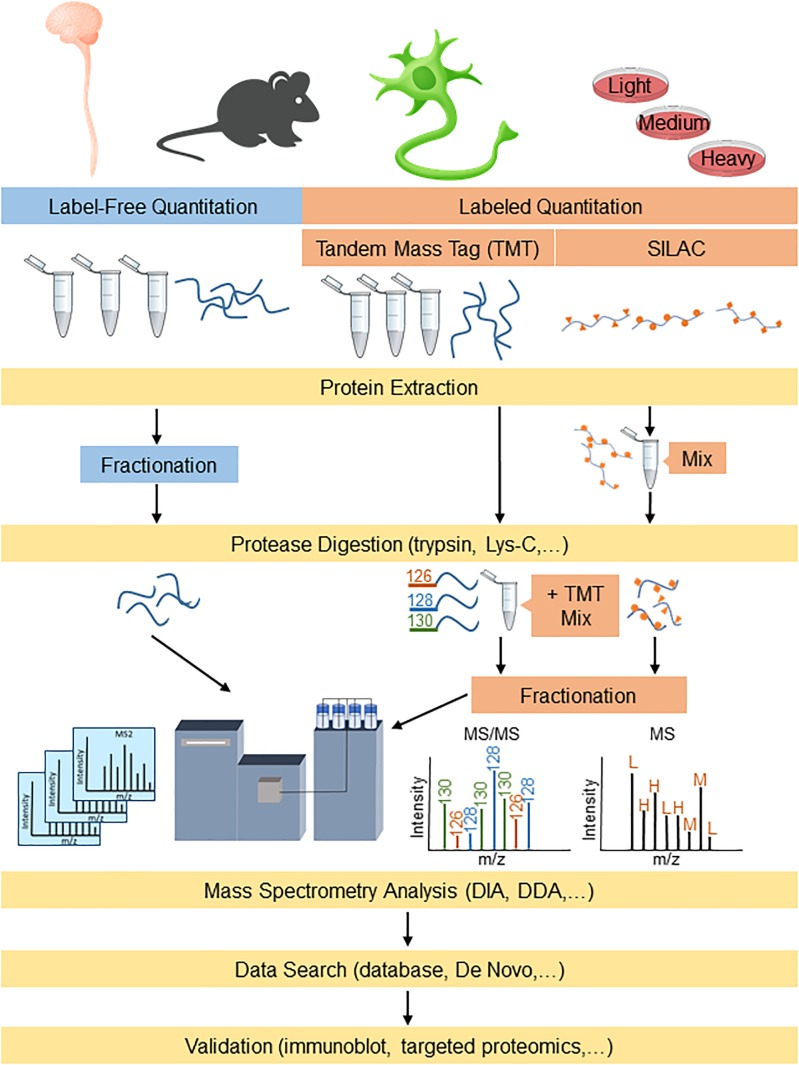
Proteomics workflow for label-free and labeling quantitation of proteins from complex mixtures relevant to understanding ALS and FTD. Protein samples derived from models/human tissue can either be labeled for targeted proteomics or analyzed label-free for broader detection. DDA, data-dependent acquisition; DIA, data-independent acquisition; and SILAC, stable isotope labeling by amino acids in cell culture.

Proteomic-based platforms are becoming increasingly powerful to identify both potential disease mechanisms as well as disease biomarkers. Proteomics involves using highly complex protein screening technology for biological understanding on a wide-scale level. This information can then be used in combination with genomic data to provide an understanding of the fundamental biological mechanisms underlying neurodegenerative diseases. The emergence of newer sophisticated mass spectrometry (MS) technology in the past decade, with higher resolution and faster scan rates, has enabled smoother and quicker identification of highly complex proteomes with shorter analysis periods (Chapman et al., 2014). The typical sample preparation procedures for proteomics analysis involves digesting proteins into peptides using a protease (e.g., trypsin and/or Lys-C) followed by reverse phase C_18_ liquid chromatographic separation and analysis by mass spectrometry (LC-MS/MS) (Tholey and Becker, 2017). The peptides that are eluted from the C_18_ column are directly ionized into the mass spectrometer for analysis, where they can be fragmented (MS/MS) further for peptide identification. The data generated by MS can be searched using algorithms that incorporate protein databases to generate lists of identified proteins. The two major streams for quantitative proteomics that are widely used are label-free and labeled quantitation (Figure 1). Both methods are used for the identification and quantitation of protein components between altered physiological states such as those in ALS and FTD, to differentiate their cellular and molecular mechanisms.

### Label-Free Quantitation Proteomics Studies of ALS and FTD

Label-free quantitation (LFQ) can use either data-dependent or data-independent acquisition (DIA) analyses. Data-dependent acquisition quantitation can be performed on either spectral counts or spectrometric signal intensity of product ions from selected precursors generated by the mass spectrometer (Yu F. et al., 2016). Recently, LFQ has been more frequently adopted for biomarker discovery studies since it is less expensive than labeled methods and allows comparative analysis of large groups of samples. A comprehensive review of the technical aspects of label-free quantitative proteomic approaches is available (Neilson et al., 2011). LFQ proteomics can also be used as an unbiased approach to characterize changes to a human proteome at a pathway level. As an example, amongst the identification of hundreds of proteins, overlapping mitochondrial and metabolic pathway alterations have been identified from samples of both human ALS and FTD brain and spinal cord samples, highlighting the dysfunctional similarities between these diseases (Iridoy et al., 2018). Similar analysis of brain tissues has provided insight into the various subtypes of ALS and FTD, distinguishing between them by highlighting differences in levels of numerous proteins as well as differences in protein aggregate assembly, distribution and morphology (Umoh et al., 2018; Laferriere et al., 2019). A sequential biochemical extraction technique to purify detergent-insoluble aggregated proteins resulted in enrichment of phosphorylated TDP-43 and identification of low-solubility proteins associated with TDP-43 pathology (Laferriere et al., 2019). Subsequent MS of these enriched proteins determined distinct patterns of enrichment amongst ALS (23 proteins) and subtypes of FTLD (FTLD-A: 8 proteins; FTLD-C: 10 proteins), providing insights into potential causes of disease heterogeneity (Laferriere et al., 2019). A summary of studies to investigate the mechanisms of ALS and FTD that have used human brain and spinal cord samples for LFQ are presented in Table 1.

**Table 1:** Human tissue proteomics studies using label-free techniques for mechanistic insight into ALS and FTD.

Sample	Summary	Remarks	References
ALS (TDP-43+) and FTLD-TDP brain	Isolation of phosphorylated TDP-43 with numerous other insoluble proteins, which differentiate ALS and FTLD-TDP subtypes	TDP-43 proteinopathy variation may stem from alternate pathological TDP-43 conformations	Laferriere et al., 2019
ALS (TDP-43+) and FTLD-TDP spinal cord and frontal cortex	281 proteins aaa/↓ in ALS and 52 ↑/↓ in FTLD-TDP (33 proteins overlap)	ALS and FTD share molecular alterations including mitochondrial and metabolic impairment	Iridoy et al., 2018
ALS and FTLD-TDP brain	Identified 15 modules of co-regulated proteins (8 significantly different across subtypes of ALS and FTLD-TDP)	Subtypes of FTLD-TDP and ALS differentiated by insoluble protein proteomic signature, possibly reflecting common/differing mechanisms	Umoh et al., 2018
ALS brain	Phosphorylation, deamidation and cleavage sites of TDP-43 almost all in the glycine-rich C-terminus	Modifications of TDP-43 may affect molecular pathways in disease	Kametani et al., 2016
ALS frontal cortex	Fungal antigens in human ALS brain within cytoplasmic structures	Fungal infection may occur in ALS	Alonso et al., 2015
ALS spinal cord	Altered detergent-insoluble protein acetylation, including GFAP, in ALS	Dysregulation of protein acetylation may be involved in ALS pathology	Liu et al., 2013
FTD frontal cortex	↓ C9ORF72 long protein isoform in disease	Novel method for quantifying C9ORF72 isoforms	Viode et al., 2018
FTLD-TDP brain	↑/↓ 50 proteins FTLD-TDP, including TDP-43 and septin 11	Identified and characterized enriched proteins in the detergent-insoluble fraction	Gozal et al., 2011b
FTLD-TDP hippocampus	↑ 54 proteins (including TDP-43) and ↓ 19 proteins in FTLD-TDP laser-captured dentate gyrus neurons	Potential pathology-associated proteins identified	Gozal et al., 2011a
FTD, PiD, CBD, intronic tau mutation and PSP brain	C-terminus of tau is protease-resistant, cleavage pattern may differentiate subtypes of FTD and AD	Analysis of tau C-terminus in FTD potentially useful in classifying disease subtypes	Taniguchi-Watanabe et al., 2016
sALS spinal cord	21 proteins ↑/↓, ↑ mitochondrial metabolism proteins, ↓ ATP5D and calmodulin	Synaptic mitochondrial changes potentially involved in ALS	Engelen-Lee et al., 2017

*↑ denotes increase; ↓ denotes decrease; AD, Alzheimer’s disease; ATP5D, mitochondrial membrane ATP synthase; CBD, corticobasal degeneration; FTD, frontotemporal dementia; FTLD, frontotemporal lobar degeneration; GFAP, glial fibrillary acidic protein; PiD, Pick’s disease; PSP, progressive supranuclear palsy; sALS, sporadic amyotrophic lateral sclerosis; and TDP-43, TAR DNA binding protein of 43 kDa.*

LFQ proteomics has also been applied to animal models, for example identifying interactors of misfolded SOD1 in the spinal cord of SOD1^G93A^ mice over three pre-symptomatic time points in disease (Ruegsegger et al., 2016). Only 5 identified proteins were common across these groups with high confidence, including HSPA8. The bulk of proteins found to interact with misfolded SOD1 were identified at the latest time point (Ruegsegger et al., 2016). Of all mutant SOD1 interactors, the activity of Na^+^/K^+^ATPase-α3 was decreased and exhibited higher levels of protein expression in high-vulnerability motor neurons (Ruegsegger et al., 2016).

Mutations in cyclin F (*CCNF*^S621G^), which cause rare familial cases of ALS/FTD, have also been studied by overexpression in neuronal cell lines and zebrafish to investigate the effects and mechanisms of ALS/FTD (Williams et al., 2016; Hogan et al., 2017; Lee A. et al., 2018). Hundreds of proteins were increased or decreased upon expression of disease-linked mutant cyclin F protein (Lee A. et al., 2018). The differentially expressed proteins clustered to cell pathways involved with cellular survival and toxicity, and predicted activation of caspase-3 mediated cell death (Hogan et al., 2017). Studies such as these that combine analysis of different models, such as cells and *in vivo* systems, using both proteomics and complementary validation approaches offer the best approach to identify biologically relevant changes. The selection of proteins for validation is often based on the statistically significant findings from the proteomic analysis as well as previously published work in the literature, however, pathway-level approaches for analysis of large datasets are often more informative than extensive analysis of individual proteins. A summary of ALS and FTD studies that have used animal models for LFQ are presented in Table 2.

**Table 2:** Animal model proteomics studies using label-free techniques for mechanistic insight into ALS and FTD.

Sample	Summary	Remarks	References
*Grn^-/-^*, *TMEM106B^-/-^* mouse brain	↑ 17 and ↓ 2 lysosomal proteins with Grn*^-^*^/^*^-^*, phenotype normalized by Tmem106b*^-^*^/^*^-^*	Alterations in lysosomal function may contribute to risk of FTD	Klein et al., 2017
Non-transgenic mouse cortex	IP-MS of TDP-43 suggests interactions with mitochondrial proteins, including PHB2	Normal function of TDP-43 may be related to mitochondria	Davis et al., 2018
rTg4510 tau mice (P301L 4R0N) brain	↑ nucleotide-binding proteins ↓ RNA binding and ribonucleoproteins in tauopathy	Identified proteins that co-localize with tau inclusions and changes in expression	Maziuk et al., 2018
SOD1^G93A^ mouse brain and spinal cord EVs	Only 1 protein ↑/↓ in SOD1^G93A^ EVs (↓ MOG)	EVs may originate from astrocytes and contain RBPs, but little change in disease	Silverman et al., 2019
SOD1^G93A^ mouse muscle tissue	ER stress response is activated in skeletal muscle of SOD1 mice ↑ CHOP, BiP and PERK	ER stress leads to reduced protein translation, involvement in muscle atrophy and weakness seen in ALS	Chen et al., 2015
SOD1^G93A^ mouse spinal cord	↓ VGF peptides, similar findings in ALS plasma	Selective depletion of VGF fragments may be involved in disease etiology	Brancia et al., 2016
SOD1^G93A^ mouse spinal cord	67 ↑/↓ lipid raft proteins, involved in vesicular transport, neurotransmitter synthesis/release, cytoskeletal organization and metabolism	Lipid raft protein changes in ALS may affect vesicular trafficking, neurotransmitter signaling and cytoskeleton	Zhai et al., 2009
SOD1^G93A^ mouse ventral horn spinal cord	IP-MS of interactors of misfolded SOD1 (three time points), primarily chaperones, transporters and hydrolases (notably HSPA8, Na^+^/K^+^ATPase-α3)	Na^+^/K^+^ATPase-α3 levels are high in vulnerable MNs, and expression may modulate disease pathology and phenotype	Ruegsegger et al., 2016
SOD1^G93A^ rat spinal cord mitochondria	↑ 33 proteins and ↓ 21 proteins, mostly involved in complex I and mitochondrial protein import	Mitochondrial protein alterations and ↓ protein import may contribute to mutant SOD1-associated mitochondrial deficits	Li et al., 2010
SOD1^WT or G93A or G85R^ mouse spinal cord	Enriched detergent-insoluble proteins, including VILIP-1	VILIP-1 may affect oxidation status and calcium levels in ALS	Liebl et al., 2014
Squid axoplasm combined with SOD1^G85R^ protein	Addition of HSP110 to mutant SOD1 affected axoplasm rescued the transport defect and phosphorylation of p38 seen in pathology	Highlights potential of HSP110 in association with HSC70 as a mediator of protein disaggregation for mutant SOD1 in ALS	Song et al., 2013
Synaptoneurosomes from rat brain	IP-MS of Chmp2b complexes, identifying association with ESCRT-III and post-synaptic proteins	Chmp2b is part of a stable complex that regulates synaptic plasticity, potentially relevant to FTD mechanisms	Chassefeyre et al., 2015
Znf179^-/-^ mouse hippocampus	IP-MS of Znf179/RNF112 identifies interaction with TDP-43, TDP-43 is polyubiquitinated by E3 ligase function of RNF112	↑ ubiquitination of TDP-43 ↑ degradation, loss of RNF112 in ALS may cause TDP-43 aggregation and pathology	Lee Y.C. et al., 2018

*↑, denotes increase; ↓, denotes decrease; ALS, amyotrophic lateral sclerosis; BiP, Endoplasmic reticulum chaperone BiP; CacyBP, calcyclin-binding protein; Chmp2b, charged multivescular body protein 2b; CHOP, DNA-damage-inducible transcript/CCAAT/enhancer-binding protein; ER, endoplasmic reticulum; ESCRT-III, endosomal sorting complex required for transport; EVs, extracellular vesicles; FTD, frontotemporal dementia; FTLD, frontotemporal lobar degeneration; HSC70, heat shock cognate 71 kDa; HSP110, heat shock protein 110; IP-MS, immunoprecipitation mass spectrometry; KO, knockout; MFN2, mitofusin 2; PERK, PKR-like endoplasmic reticulum kinase; PGRN, progranulin; PHB2, prohibitin 2; PMPCA, mitochondrial-processing peptidase subunit alpha; RBPs, RNA-binding proteins; RNF112/ZNF179, RING finger protein 112; SOD1, superoxide dismutase 1; TDP-43, TAR DNA binding protein of 43 kDa; TMEM106B, transmembrane protein 106B; VGF, neurosecretory protein VGF; and VILIP-1, Visinin-like protein 1.*

The majority of the previous investigative studies have used heterogeneous samples, which poses a limitation. Rather than representing proteomes from only a specific cell type of interest, they largely consist of populations of homogenized and variable cell types. To account for this, reemergence of laser-capture microdissection of tissues has been used to isolate distinct cell-types, and with recent advances in MS technologies this can now be successfully applied to proteomics studies of neurons from brain samples (Aring et al., 2018). Newer technology that is highly sensitive and specific can thus generate a greater number of protein hits from small amounts of extracted sample, and this offers opportunities for future studies of both model systems and human ALS and FTD samples.

In addition to studies of tissue samples, much proteomics research has been applied to understand ALS and FTD pathogenesis using cell culture models. This approach, which usually makes use of homogenous populations of cells such as immortalized cell lines, removes the confound of studying mixed samples that is often inherent using tissue samples and biofluids. Expression of disease-associated proteins, including mutant SOD1, TDP-43, and tau, recapitulate some features of disease in cell models, such as protein aggregation, inclusion formation and cellular toxicity (Gauthier-Kemper et al., 2011; Cohen et al., 2015; Celona et al., 2017; Monahan et al., 2017; Wang et al., 2017). Proteomics studies of these models have been used to identify proteins that co-aggregate or interact with the known pathological proteins, which may provide insight into disease pathogenesis. Identification of proteins associated with disease pathology may also help reveal proteins of potential therapeutic use if, for example, those proteins are able to modulate protein aggregation or toxicity (Celona et al., 2017; Mallik et al., 2018). A summary of ALS and FTD studies that have used cell models for LFQ are presented in Table 3.

**Table 3:** Cell and *in vitro* model proteomics studies using label-free techniques for mechanistic insight into ALS and FTD.

Sample	Summary	Remarks	References
Differentiated C2C12 mouse myoblasts	Detected muscle specific kinase activation via phosphorylation, which preserves innervation of neuromuscular junctions	Innervation of neuromuscular junctions insufficient, however, could potentially be used as an adjuvant therapy	Sengupta-Ghosh et al., 2019
HEK239T cells and rat primary cortical neurons infected with GR_149_ or PR_175_	Poly-GR/PR interactome identified RNA-binding proteins (many low-complexity domains), cytoplasmic/mitochondrial ribosomes components, stress granules and splicing factors	Sequestration of ribosomes via interactions with poly-GR/PRs would potentially impair protein translation in FTD pathology	Hartmann et al., 2018
HEK293 and N2A cells transfected with cyclin F^WT or S621G^	Identified 7 phosphorylation sites on cyclin F	Cyclin F S621 phosphorylation by CK2 regulates Lys48-specific E3 ligase activity	Lee A. et al., 2017
HEK293 cells expressing RBM45	Identified 132 protein-protein interactors of RBM45	RBM45 associates with enriched proteins involved in nuclear RNA processing: TDP-43, Matrin-3, hnRNP-A1 and FUS	Li et al., 2016
HEK293A cells expressing TDP-43^ΔNLS and 2KQ^ and CBP	Identified TDP-43 acetylation sites K145/192	TDP-43 modulation via acetylation could potentially be used therapeutically	Cohen et al., 2015
HEK293E cells expressing TDP-43^192-414 or^ ^ΔNLS or WT^	Removal of 4 lysine ubiquitination sites in CTF TDP-43 = ubiquitination suppression	Indicates interplay between ubiquitination and phosphorylation of TDP-43 in ALS and FTD pathology	Hans et al., 2018
HEK293T and H4 cells treated with various drugs	Identified 28 phosphorylation sites within FUS’s prion-like domain, following DNA-damaging stress	Multiphosphorylation of these sites does not cause cytoplasmic localization	Rhoads et al., 2018
HEK293T cells expressing C9ORF72 DPRs	Interactome of DPRs: RNA-binding proteins and proteins with low complexity sequence domains	DPRs altered phase separation of low complexity domain proteins, suggesting possible mechanism involved in pathogenesis	Lee et al., 2016
HEK293T cells expressing FUS^P525L^	Mutant FUS ↓ interactions with many metabolic enzymes. Novel interactions between FUS and VCP, PSF, UBA1 and PSMD12. FUS accumulation = ↓ ATP levels and ↑ poly-ubiquitinated proteins	Defective energy metabolism and protein degradation arise as a result of FUS accumulating and interacting with key regulators	Wang et al., 2015
HEK293T cells expressing FUS^R521G or P525L^	FUS interacting proteins = fALS implicated proteins hnRNPA1 and Matrin-3	Potential common pathogenic roles between FUS-ALS and fALS. FUS present in exosomes suggesting contribution to cell-to-cell transmission/spread. Interactors also sequestered into inclusions	Kamelgarn et al., 2016
HEK293T cells expressing mPGRN-HA	PGRN interacts with a network of ER chaperones such as BiP, calreticulin, GRP94 and PDI family proteins	PGRN is a substrate of several PDI proteins and ER chaperone network control could be a therapeutic target	Almeida et al., 2011
HEK293T cells transfected with Zfp106	Zfp106 interacts with hexanucleotide repeat (GGGGCC) RNA-binding protein, other RNA-binding proteins: TDP-43 and FUS. Zfp106 KO mice develop motor neuron degeneration. Zfp106 suppresses neurotoxicity in *Drosophila* C9orf72 ALS model	Importance and role of Zfp106 in ALS pathology	Celona et al., 2017
HEK293T cells treated with various drugs	Identified 17 phosphorylation sites within FUS low-complexity domain	Phosphorylated/phosphomimetic FUS reduces aggregation, propensity to aggregate, ameliorates cytotoxicity and disrupt phase separation	Monahan et al., 2017
HEL293FT cells expressing C9ORF72 DPRs	Co-aggregators of poly-GA = Unc119, soluble Unc119 ↓ in poly-GA expressing neurons	Loss of function of Unc119 in neurons with DPR-type pathology as seen in C9orf72 ALS/FTD	May et al., 2014
HeLa cells expressing C9ORF72 DPRs	Arginine-rich DPRs undergo liquid-liquid phase separation and induce this effect on proteins involved in RNA and stress granule metabolism	Arginine-rich DPRs derived from C9ORF72 repeat expansions play an important role in the pathogenesis of ALS/FTD	Boeynaems et al., 2017
HeLa cells expressing FUS	FUS forms liquid-like compartments under stress that are crucial for its role in ALS	Propensity for aggregation vs functionality of FUS action in liquid-compartments found in disease	Patel et al., 2015
*In vitro* (PGRN)	Cathepsin L cleaves intracellular PGRN	Cathepsin L identified as a key intracellular lysosomal protease, therefore demonstrating link between lysosomal dysfunction and FTLD	Lee C.W. et al., 2017
*In vitro* (SOD1)	SOD1^G37R^ did not have the same propensity to aggregate as SOD1^G93A^ and SOD1^V 148G^, however, still formed oligomeric aggregates	Slow disease progression in SOD1^G37R^ patients is due to structural limitations associated with the arginine substitution at residue 37	McAlary et al., 2016
*In vitro* (SOD1)	Naringin is a strong native interactor of SOD1, demonstrated to stabilize SOD1 dimers and inhibit aggregation	Analytical method for studying interactions between proteins and drug-like molecules, identifying role of naringin	Zhuang et al., 2016
*In vitro* (tau)	K225,240,257,311,383 residues in tau involved in crosslinking to K336,338 in α-tubulin	Identified how tau stabilizes microtubules through identifying sites of interface	Kadavath et al., 2015
iPSC-derived motor neurons expressing C9ORF72 DPRs and *Drosophila* brain	Arginine DPRs interact with ribosomal proteins, expression of eIF_1_A rescued DPR-induced toxicity	Repression of protein translation is involved in C9orf72 hexanucleotide-repeat induced neurodegeneration	Moens et al., 2019
Mouse primary hippocampal neurons expressing scrambled shRNA FUS	Identified PSD-95 interacting proteins: ↓ SynGAP with FUS depletion. FUS, ELAV1 and ELAV4 exert a level of control on SynGAP mRNA stability	FUS depleted dendritic spines associated with internalization of PSD-95	Yokoi et al., 2017
N2a cells expressing C9ORF72	Interactors of C9ORF72 = UBQLN2, hnRNPA2/B1, hnRNPA1 and actin. Colocalization with RAB7 and RAB11 suggests dysregulation of trafficking	Demonstrates the role of C9ORF72 in RAB-mediated trafficking	Farg et al., 2014
N2a cells expressing TDP-43 and CTF	Interactome of TDP-43 using BioID identified 254 proteins vs 389 in CTF, many involved in mRNA processing	TDP-43 aggregate associates were involved in nuclear pore complex and transport machinery	Chou et al., 2018
NSC-34 cells expressing C9ORF72 DPRs	Poly-PR peptides interact with mRNA-binding proteins, ribosomal proteins, translation initiation and elongation factors	Alterations via DPRs are potential therapeutic targets and are involved in neurotoxicity	Kanekura et al., 2016
PC12 cells expressing SOD1^G93A^	PSMC1, PSMC4 and TCP-1 activated by pyrazolones in the absence of exogenous proteasome inhibitor	In the absence of the heat shock response, pyrazolones enhance proteasomal activation and could be a potential therapeutic target	Trippier et al., 2014
S2 cells expressing Xrp1^Short or Long^ and actin5C-GAL4	↑ Xrp1 expression in caz mutants, interactors are involved in gene expression regulation	Caz is the ortholog of human FET proteins FUS, EWSR1, and TAF15, all of which implicated in ALS and FTD, dysregulation of gene repair implicated	Mallik et al., 2018
SH-SY5Y cells knockdown TDP-43	↓ RanBP1, Dnmt3a and CgB in TDP-43 knockdown. ↓ RanBP1 = ↑ transportin 1	TDP-43 mediates RNA metabolism and intracellular transport	Stalekar et al., 2015
SKNBE2 cells expressing tau	AnxA2 interacts with tau^WT^ but not mutant tau^R406W^	Tau^R406W^ mechanism involves impaired membrane binding due to functional interaction with AnxA2	Gauthier-Kemper et al., 2011
SOD1 isolated from yeast	Low molecular weight fractionated SOD1 does not appear to be post-translational modified compared to high molecular weight SOD1, which is oxidized at residues C146 and H71	Crucial for SOD1 structure, suggesting a role of oxidative damage for protein misfolding	Martins and English, 2014
SOD1^WT or G37R or L38V or G41D or^ ^G93A or G93S or D101N^ isolated from yeast	Structure of SOD1 amyloid fibrils and mutants demonstrated that fibrils protected the N-terminus from digestion via proteases	SOD1 and mutants fibrillate through the N-terminal fragment, highlighting potential ALS implications	Chan et al., 2013

*↑, denotes increase; ↓, denotes decrease; AnxA2, Annexin A2; caz, RNA-binding protein cabeza; CgB, chorionic gonadotropin beta; CK2, casein kinase II subunit alpha; CTF, C-terminal fragment; Dnmt3a, DNA (cytosine-5)-methyltransferase 3A; DPR, dipeptide repeat; EIF1A, eukaryotic translation initiation factor 1A; ELAV1/4, embryonic lethal abnormal vision protein 1/4; EWSR1, RNA-binding protein EWS; FUS, fused-in-sarcoma; GFAP, glial fibrillary acidic protein; GR, glycine arginine; GRP94, 94 kDa glucose regulated protein; hnRNPA1, heterogeneous nuclear ribonucleoprotein A1; iPSC, induced pluripotent stem cell; MTO, methanethiol oxidase; PDI, protein disulphide isomerase; PIN1, peptidyl-prolyl *cis-trans* isomerase NIMA-interacting 1; PR, proline arginine; PSD-95, post-synaptic density protein 95; PSF, polypyrimidine tract-binding protein-associated-splicing factor; PSMC1/4, 26S proteasome regulatory subunit 1/4; PSMD12, 26S proteasome non-ATPase regulatory subunit 12; RAB7/11, Ras-related protein Rab-7/11; RanBP1, Ran-binding protein 1; RBM45, RNA binding motif protein 45; ROCK2, rho-associated protein kinase 2; SOD1, superoxide dismutase 1; SynGAP, Ras/Rap GTPase-activating protein SynGAP; TAG15, TATA binding associated factor 15; TCP-1, T-complex protein 1; TDP-43, TAR DNA-binding protein of 43 kDa; UBA1, ubiquitin-like modifier-activating enzyme 1; UBQLN2, ubiquilin-2; Unc119, protein unc-119 homolog A; VCP, vasolin-containing protein; and Zfp106, zinc finger protein 106.*

The main limitations of LFQ using data-dependent acquisition is the generally low proteome coverage and low sensitivity, since many low intensity ions (often from low abundance proteins or poorly ionized peptides) are missed in precursor ion selection. In order to improve this coverage, additional sample prefractionation steps (such as strong cation exchange or basic reverse phase chromatography) can be used to reduce the complexity of the sample to be analyzed by MS. A recent LFQ approach that is being more widely adopted relies on data independent quantitation with methods such as Sequential Window Acquisition of all Theoretical Mass Spectra (SWATH) that attempt to circumvent some of these issues associated with analyzing complex samples by data-dependent acquisition.

SWATH, or data-independent acquisition (DIA), acquires data by cycling through predefined sequential windows of a chromatographic elution range generating a larger number of identified proteins from a complex mixture (Gillet et al., 2012). Recently, SWATH has been used to identify differences in blood samples between 42 ALS patients and 18 healthy controls, which revealed a panel of novel potential biomarkers for diagnosis and use in clinical trials (Xu et al., 2018). This study found significant differences in the expression of 30 proteins that varied in ALS patients with or without cognitive impairments. This study highlights the potential of DIA methodologies, such as SWATH, to discover markers in biofluids that may have further utility for clinical use that offers promise to provide further advances in ALS and FTD studies. A comprehensive review of the technical aspects of SWATH is discussed elsewhere (Ludwig et al., 2018).

Matrix-assisted laser desorption ionization (MALDI) is an alternative method to introduce a sample into a MS. This differs from electro-spray ionization (ESI) through focusing laser energy at a matrix-embedded sample for low fragmentation and reduced multi-charged ion states (Nadler et al., 2017). Typically ESI dominates the literature as the intermediate in LC-MS, used in all the studies presented in Tables 1–3. There are limitations to each technique, which are discussed elsewhere (Nadler et al., 2017), and the choice of technique generally depends on the biological question. For example, in ALS and FTD studies, MALDI-MS has been successfully applied to identify increased levels of ubiquitin carboxy-terminal hydrolase L1 (UCHL1) in FTLD-tau cortex (Schweitzer et al., 2006) as well as increased carbonylation of UCHL1 in the spinal cord of SOD1 mice (Poon et al., 2005). Together these findings have highlighted the roles of the oxidative stress response and ubiquitin-mediated degradation in ALS and FTD. MALDI-MS has also been used to identify increased levels of proprotein convertase 1 inhibitor (ProSAAS) in FTD CSF (Davidsson et al., 2002) and potentially pathologically involved ProSAAS N-terminal fragments in the temporal lobe of FTD samples (Kikuchi et al., 2003). More recently, MALDI-MS has been used to identify interactions between Staufen1 and dynein, mediated via protein phosphatase 1-beta, implicating a role of Staufen1 in regulating mRNA localization in ALS (Gershoni-Emek et al., 2016). This has been reinforced through a described link between Staufen1 RNA stress granules and autophagy through interaction with ataxin-2 (Paul et al., 2018). A summary of ALS and FTD studies that have used MALDI-MS is presented in Table 4.

**Table 4:** MALDI-MS proteomics studies for mechanistic insight into ALS and FTD.

Sample	Summary	Remarks	References
**Human samples**		
ALS spinal cord	CA-I is biotinylated alongside SOD1 + immunoreactive to a SOD1 antibody	Suggests altered CO_2_ transport and cellular pH homeostasis	Liu et al., 2010
sALS spinal cord	18 proteins ↑/↓, GFAP = most abundant	Proteins involved in apoptosis and cytoskeleton stabilization	Ekegren et al., 2006
FTLD frontal cortex	24 proteins ↑/↓ = UCHL1 and oxidative stress proteins	Ubiquitin-mediated degradation and oxidative stress response altered	Schweitzer et al., 2006
FTLD-tau (Pick disease) brain	↑ GFAP with glycoxidation and lipoxidation	GFAP = target of oxidative damage	Muntane et al., 2006
FTD temporal lobe	N-terminal fragment ProSAAS enriched in tauopathies	ProSAAS is an inhibitor of neuroendocrine peptide processing - enrichment may cause functional perturbation	Kikuchi et al., 2003
FTD CSF	↑/↓ ProSAAS, PEDF, RBP, apoE, HP, and ALB	Comparative proteomics to establish pathophysiological mechanisms	Davidsson et al., 2002
sALS spinal cord	↑ Detergent-insoluble proteins (ACO2, HSC70, and PPIase A) + intermediate filaments, chaperones and mitochondrial proteins, some tyrosine-nitration	Aggregation-prone proteins and nitrative stress contribution to inclusion pathology	Basso et al., 2009
**Animal models**		
SOD1^G93A^ mouse synaptic fractions	STAU1+dynein interactions via PP1B	STAU1 regulates mRNA localization in axons and synapses. Disrupted = toxicity	Gershoni-Emek et al., 2016
SOD1^G93A^ mouse facial and trigeminal nuclei	↑/↓ various proteins, ↑RPS19	Proteins contributing to pathology via comparative brain region proteomics	Acquadro et al., 2014
SOD1^H46R/H48Q^ mouse spinal cord	Association between SOD1 surface hydrophobicity SOD1 and conformations	HSF1 activation may mitigate ALS pathology	Lin et al., 2013
SOD1^G93A^ ^and G127X^ mouse spinal cord	Mutant SOD1 interactors = chaperones, HSC70 abundant	Chaperone depletion is not involved in SOD1 mutations of ALS	Zetterstrom et al., 2011
SOD1^G85R and G93A^ mouse eMNs	↑ CRMP4a = axonal degeneration and MN cell death ↓ CRMP4a protective	CRMP4a pathologically involved in ALS	Duplan et al., 2010
SOD1^G127X^ mouse spinal cord	54 proteins ↑/↓ = oxidative stress, mitochondrial, cellular assembly/organization and protein degradation	Altered pathways may contribute to disease	Bergemalm et al., 2009
SOD1^G93A^ mice spinal cord	↑/↓ proteins = mitochondrial dysfunction, aggregation and stress response	Potential presymptomatic targets	Massignan et al., 2007
SOD1^G93A^ mouse spinal cord	↑ Carbonylation of SOD1, TCTP, UCHL1, and CRYAB	Oxidative modification contributing factor to ALS	Poon et al., 2005
SOD1^G93A^ mouse spinal cord	Peroxidation of DRP-2, HSP70, and ENO1	Supports oxidative stress as a major pathological mechanism	Perluigi et al., 2005
hTau40^P301L^ mouse brain	↓ Complex I activity, ↑ antioxidant enzymes, altered lipid peroxidation	Tau pathology involves mitochondrial and oxidative stress	David et al., 2005
**Cell models and in vitro studies**		
N2A cells expressing ATXN2, FUS, C9ORF72, OPTN, TDP-43, and UBLQN2 WT/mutants	Interactome of ATXN2, C9ORF72, FUS, OPTN, TDP-43, and UBQLN2 (hundreds of proteins)	Strong interactome overlap for ATXN2, FUS, and TDP-43 distinct from OPTN and UBQLN2	Blokhuis et al., 2016
C4F6 hybridoma cells expressing SOD1 mutants	D92/D96 important for SOD1-C4F6 antibody interaction	C4F6 antibody epitope in SOD1 is a potential therapeutic target	Rotunno et al., 2014
COS7 cells expressing PGRN	4 N-glycosylation sites of PGRN	PGRN glycosylation may contribute to disease	Songsrirote et al., 2010
N2A cells treated with cadmium	Cadmium = ↑/↓ proteins = cellular structure, stress, chaperones, cell death/survival and ROS	Heavy metals suppress function of SOD1	Huang et al., 2006
NSC-34 cells expressing SOD1^G93A^	170 proteins, ↑/↓ = mitochondrial, membrane transport, apoptosis, respiratory chain and chaperones	Mitochondrial protein changes = evidence for mitochondrial dysfunction	Fukada et al., 2004
SOD1^WT or N26D/N131D/N139D^ isolated from yeast	Deamidation mimic mutant SOD1 aggregated into amyloid fibrils faster than WT	Deamidation may be involved in SOD1 pathology	Shi et al., 2013b
*In vitro* (tau)	Acetylated tau prevents degradation of phosphorylated tau	Tau acetylation may be a therapeutic target	Min et al., 2010

*↑, denotes increase; ↓, denotes decrease; ACO2, aconitase; ALB, albumin; apoE, apolipoprotein E; ATXN2, ataxin-2; C4F6; CA-I, carbonic anhydrase I; CRMP4a, collapsin response mediator protein 4a; CRYAB, alpha-crystallin B chain; CSF, cerebrospinal fluid; DRP-2, dystrophin-related protein 2; eMNs, embryonic motor neurons; ENO1, alpha-enolase; FTD, frontotemporal dementia; FTLD, frontotemporal lobar degeneration; FUS, fused-in sarcoma; GFAP, glial fibrillary acidic protein; HP, haptoglobin; HSC70, heat shock cognate 71 kDA; HSP70, heat shock 70 kDa; OPTN, optineurin; PEDF, pigment epithelium-derived factor; PGRN, progranulin; PP1B, protein phosphatase 1-beta; PPIase A, peptidyl-prolyl cis-trans isomerase A; ProSAAS, proprotein convertase 1 inhibitor; RBP, retinol binding protein; ROS, reactive oxygen species; RPS19, 40S ribosomal protein S19; sALS, sporadic amyotrophic lateral sclerosis; STAU1, Staufen1; TCTP, translationally controlled tumor protein; TDP-43, TAR DNA-binding protein of 43 kDa; UBQLN2, ubiquilin-2; and UCHL1, ubiquitin carboxy-terminal hydrolase L1.*

### Quantitation via Labeling Proteomics Approaches for ALS and FTD

Labeled-based approaches have considerably higher quantitation accuracy in exchange for lower proteome coverage compared to LFQ (Megger et al., 2014). The labeling techniques involve the introduction of stable isotope labels on the proteins or peptides, which allow the mass spectrometer to distinguish between identical peptides from different samples within a mixture. Quantitative labeling methods can also be used for protein-protein interactions (PPIs) and post-translational modifications (PTM) analyses which are discussed below (Sullivan et al., 2016; Russell et al., 2017). A comprehensive review of the technical aspects of labeled-based proteomic approaches is available (Lindemann et al., 2017).

#### Stable Isotope Labeling by Amino Acids in Cell Culture (SILAC)

Stable Isotope Labeling by Amino acids in Cell culture (SILAC) labeling is achieved by growing cells in culture with growth medium containing different isotopically labeled amino acids that are incorporated during protein synthesis (Ong et al., 2002). The protein lysates from different isotope-labeled cell culture are pooled together in equal amounts and prepared as one grouped sample to decrease sample preparation variability. High resolution mass spectrometers can analyze and resolve the different precursor (peptide) ions within an experiment and detect the signal intensities of the labeled peptides. The intensities of the precursor ions are used as a measure of the relative abundance of the protein within each sample. One limitation of SILAC, however, is the relatively small number of labeled amino acid combinations [maximum reported to date is 6-plex (Wang et al., 2013)] that can be used in one experiment, which limits the number of comparisons.

The versatility of SILAC can extend to the generation of an internal standard in a cell-line to quantify low abundance proteins in tissue (known as “Super SILAC”). This was applied to investigate the accumulated proteins in detergent-insoluble brain lysates from four control and four FTLD brain samples, with comparison to a HEK293 standard (Seyfried et al., 2012). A summary of ALS and FTD studies that have used SILAC is presented with other labeled methods in Table 5.

**Table 5:** Labeled-MS proteomics studies for mechanistic insight into ALS and FTD.

Technique	Model	Summary	Remarks	References
iTRAQ	SOD1^G93A^ mouse spinal cord	↑ 676 proteins, ↓ 480 proteins	Preliminary insight into altered proteins	Zhang J. et al., 2018
iTRAQ	Rat neonate spinal cord injected with ALS CSF	↓ 35 mitochondrial and 4 lysosomal proteins and ↑ BNIP3L	Mitochondrial and lysosomal defects involved in pathogenesis	Sharma et al., 2016
SILAC	HeLa cells expressing FUS/TLS	↑ FUS/TLS mutant interaction with SMN and ↓ interaction with U1-snRNP	Demonstrates a gain and loss of function in FUS-ALS	Sun et al., 2015
SILAC	Primary mouse astrocyte cultures	60 astrocyte proteins regulate secretion with Ang stimulation	Ang taken up by astrocytes = potential neuroprotection	Skorupa et al., 2013
SILAC	HEK-293 cells expressing TDP-43 or splice variant	35 proteins co-aggregate with TDP-43 (G3BP, PABPC1 and eIF4A1), 4 ubiquitinated sites on TDP-43 splice variant	TDP-43 aggregation partners + ubiquitination affects oligomerization	Dammer et al., 2012
SILAC and label-free	H1299 cells and HEK293T cells expressing UBXD1 or ERGIC-53	Interactome of UBXD1 includes VCP and ERGIC-53, highlighting its role in vesicle trafficking	UBXD1 involved in regulation of ERGIC-53 trafficking through its interaction with VCP	Haines et al., 2012
SILAC and label-free	HeLa cells expressing TDP-43	TDP-43 interacts with hnRNP, Drosha and FUS/TLS complexes, mutant TDP-43 ↑ FUS/TLS interactions	TDP-43 involved in mRNA processing and miRNA biogenesis, function overlap with FUS	Ling et al., 2010
TMT	C9ALS and sALS iPSCs	RNA stability/binding targets: ↑/↓ 170 altered in C9ALS and 121 in sALS	Destabilization of RNA transcripts involved in oxidative phosphorylation and ribosomal machinery	Tank et al., 2018
TMT	hESC and hESC MN cells expressing C9ORF72, immunoprecipitated	C9ORF72 stabilizes SMCR8, enables interaction with WDR41	C9ORF72+SMCR8 involved in autoimmunity and lysosomal exocytosis	Zhang Y. et al., 2018
TMT	HeLa cells expressing C9ORF72 DPR proteins	C9ORF72 PR and GR DPRs block spliceosome assembly	DPR-mediated dysfunction = Mis-spliced exons in C9ORF72	Yin et al., 2017
TMT	C57BL/6J tau KO mouse brain	Interactome of tau and isoforms – 101 proteins identified	Selective binding of proteins with specific isoforms of tau	Liu et al., 2016
TMT and iTRAQ	HEK293T and SH-SY5Y expressing various tau proteins	Tau^P301L^ disrupts interactions with heat shock, proteasome and microtubule-associated proteins	Mutant tau^P301L^ ↓ interactions with chaperones and proteasome	Gunawardana et al., 2015

*↑, denotes increase; ↓, denotes decrease; Ang, angiogenin; BNIP3L, BCL2 interacting protein 3-like; CSF, cerebrospinal fluid; DPR, dipeptide repeat; eIF4A1, eukaryotic translation initiation factor 4A1; ERGIC-53, ER-Golgi intermediate compartment 53 kDa protein; FTD, frontotemporal dementia; FUS, fused-in-sarcoma; G3BP, ras GTPase-activating protein; GR, glycine arginine; hESC, human embryonic stem cell; iTRAQ, isobaric tags for relative and absolute quantitation; KO, knockout; MN, motor neuron; PABPC1, polyadenylate-binding protein 1; PR, proline arginine; sALS, sporadic amyotrophic lateral sclerosis; SILAC, stable isotope labeling by amino acids in cell culture; SMCR8, guanine nucleotide exchange protein; SMN, survival motor neuron protein; TDP-43, TAR DNA binding protein of 43 kDa; TMT, tandem mass tag; UBXD1, UBX domain-containing protein 1; VCP, valoslin-containing protein; and WDR41, WD repeat-containing protein 41.*

#### Tandem Mass Tags (TMT)

Unlike SILAC, which incorporates isotopically labeled amino acids at the protein level, the Tandem Mass Tag (TMT) system is used to label peptides following proteolytic digestions (Thompson et al., 2003). TMT tags are covalently coupled to both N-terminal α-amino groups and 𝜀-amino groups of lysine residues (Thompson et al., 2003). Once labeled, peptide samples are pooled (commonly up to 10-plex), and subsequently fractionated and analyzed by a high-resolution mass spectrometer. The secondary detection of reporter ions (MS2) allow the peptides to be quantitated based on the TMT signal intensities, which can be extended to a third round (MS3) to decrease ratio distortion (Ting et al., 2011).

TMT-based quantitative proteomics is being increasingly applied in neuroscience, including in several investigations of proteome-wide nucleocytoplasmic changes in cell-based models of ALS. In mouse motor neuron-like NSC-34 cells overexpressing mutant hSOD1^G93A^, TMT-labeling of peptides combined with the results of RNA-Seq demonstrated impairments to nuclear and cytoplasmic transport (Kim et al., 2017). Specifically, proteins enriched in the nuclear fraction of mutant SOD1-expressing cells were related to RNA transport/processing and known Huntington’s disease/Alzheimer’s disease pathways, whilst proteins enriched in cytoplasmic fractions were involved in protein folding, aminoacyl-tRNA biosynthesis, Wnt signaling, synaptic vesicle cycle and Hippo signaling pathways (Kim et al., 2017). In another study, TMT 10-plex was used to analyze ALS patient fibroblast-derived iPSC cells to validate genome-wide RNA instability in ALS and FTD patients (Tank et al., 2018). Bromouridine tagging of RNA transcripts identified profound destabilization of ribosomal and mitochondrial transcripts, which was verified by TMT-quantitative proteomics and revealed corresponding decreases in mitochondrial components and compensatory increases in protein synthesis (Tank et al., 2018). This approach suggested that RNA instability could be a targeted effect of TDP-43 accumulation, disrupting energy production and protein synthesis, culminating in cell death (Tank et al., 2018). A summary of studies that have used various labeling-based MS techniques such as TMT for investigation of ALS and FTD is presented in Table 5.

### Protein-Protein Interactions

Interrogating PPIs in cultured cells, animal models of disease and from human tissue can provide valuable insight into mechanisms that underlie neurodegeneration. Characterizing the interactome of proteins of interest in both healthy and disease states not only reveals normal protein function but also sheds light on pathogenic changes. Characterizing PPIs also has therapeutic value since specific interactions may be amenable to therapeutic modulation.

MS-based methods enable high-throughput identification of PPIs and have been extensively used to characterize the interactomes of many proteins implicated in neurodegeneration (Hosp et al., 2015). Standard methods used to identify PPIs are based on an immunoprecipitation (IP) followed by MS; however, in recent years, proximity-ligation methods have emerged which complement standard IPs and provide additional insight into protein interactomes. These methods include proximity-dependent biotin identification (BioID) and ascorbate peroxidase (APEX)-based proximity tagging (Roux et al., 2012, 2013; Chu et al., 2017). BioID, APEX and related techniques can also be used to study interaction partners of insoluble proteins, a feature which makes these methods particularly useful when analyzing neurodegenerative diseases characterized by protein aggregation (Roux et al., 2013).

#### Immunoprecipitation and MS (IP-MS)

A standard method that is used to identify PPIs relies on maintaining the physical interaction between the interaction partners. Prior to IPs, cells are lysed in non-denaturing buffers which can maintain stable but not transient interactions. A typical process for IP-MS studies is based on antibody recognition of a protein of interest within a lysate, followed by specific isolation of the antibody (and associated proteins) using protein A or G conjugated to beads (Markham et al., 2007). Of note, this method requires that PPIs remain stable after cell lysis and that the protein complex of interest remains soluble in the chosen lysis buffers. In ALS and FTD studies, IP-MS has been used to identify interacting partners of TDP-43, revealing many proteins involved in RNA metabolism (Freibaum et al., 2010). Recently, identified interactors of RNA-binding protein 45 (RBM45), a protein that colocalizes with ALS and FTD inclusions, were associated with multiple hnRNP and EIF proteins involved in RNA metabolism, suggesting disturbance of these processes upon pathology formation in disease (Li et al., 2016). As IP-MS studies generally incorporate label-free MS they have been included amongst studies previously discussed in Tables 1–3.

#### Proximity-Ligation Methods

BioID and APEX are proximity-ligation methods which facilitate the covalent attachment of biotin onto proteins in close proximity to a protein of interest. The application of these methods for interrogating PPIs of proteins is based on the strong interaction between biotin and streptavidin (Green, 1990) and the stability of streptavidin in a large range of conditions. This includes stability in denaturing buffers containing ionic detergents such as sodium dodecylsulfate (SDS) (Sano et al., 1998) and chaotropic reagents such as urea (Kurzban et al., 1991), such that these approaches are applicable to studies of both detergent-soluble and -insoluble proteins from cells and tissues.

##### Proximity-dependent biotin identification (BioID)

Proximity-dependent biotin identification or BioID is based on the use of an engineered biotin ligase which carries an R118G mutation within its active site, effectively nullifying self-association and DNA binding (Roux et al., 2012). Normally, biotin ligase (BirA) works by converting biotin to a highly reactive biotinoyl-AMP intermediate in an ATP-dependent manner. This intermediate is then deposited onto lysine residues within the natural substrate of BirA, Biotin Carboxyl Carrier Protein (BCCP) (Cronan, 1990). Engineered biotin ligase (BirA^∗^) is also able to convert biotin to a highly reactive intermediate, however, due to this R118G mutation, the intermediate is prematurely released from the active site of BirA^∗^ and diffuses away leading to biotinylation of nearby lysine residues (Kwon et al., 2000). In this way BirA^∗^ can biotinylate lysine residues of proteins in close proximity to the protein of interest. In a typical BioID workflow, a construct encoding BirA^∗^ in frame with a protein of interest is first generated and expressed in live cells. Exogenous biotin is then added to cell culture media such that biotin can be processed by BirA^∗^ and deposited onto proteins within proximity to the fusion protein. Here, labeling typically occurs over 12–24 h to generate enough material for analysis. In addition, the half-life of the biotin intermediate is in the minutes range which results in a large labeling radius (Rhee et al., 2013). Cells can then be lysed before streptavidin-conjugated beads are used to isolate biotinylated proteins from the complex mixture. These isolated biotinylated proteins can then be identified using standard MS-based workflows. BioID approaches have only recently begun to be applied to studies to understand mechanisms of ALS and FTD and discussion continues into their use for neurodegenerative diseases (Rayner et al., 2019). Most notably, a BioID approach was used to characterize the interactome of TDP-43 and a C-terminal fragment which revealed a correlation between C-terminal fragments and nuclear pore defects (Chou et al., 2018).

In recent years, a smaller, more efficient form of the biotin ligase, termed BioID2, has been established (Kim et al., 2016). BirA^∗^ has also been modified using directed evolution which has improved the labeling kinetics of biotin ligase such that labeling can be completed in 10 min. This variant of BirA^∗^, termed “TurboID” has been implemented in live organisms including *Caenorhabditis elegans* and *Drosophila melanogaster* (Branon et al., 2018), demonstrating the versatility of this method to interrogate the interactome of proteins in both live cells and organisms. These improvements make this system a key new technology for future application to the understanding of protein interactions in neurodegeneration, using both cultured cells and animal models of disease.

##### Ascorbate peroxidase (APEX)-based proximity tagging

APEX is a 28 kDa engineered peroxidase that is derived from dimeric pea or soybean ascorbate peroxidases (Martell et al., 2012; Rhee et al., 2013). APEX can be used in a similar manner to BioID for the biotinylation of proteins in the vicinity of a protein of interest. Like BioID, constructs are first generated to encode engineered soybean peroxidase in frame with a protein of interest before the fusion protein is expressed in live cells. Notably, APEX can be used for temporally resolved proteomics as well as high-resolution microscopy, making this a flexible technique for studies of protein interactions.

Once the APEX fusion protein is expressed in live cells, exogenous biotin is added to cell culture media. For labeling of proximal proteins, hydrogen peroxide (H_2_O_2_) and biotin phenol is added to media such that APEX can catalyze a one-electron oxidization of biotin phenol to a biotinphenoxyl radical which subsequently biotinylates proteins in proximity (Lam et al., 2015). Notably, labeling kinetics of APEX is fast. The produced biotin-phenoxyl radical lasts for <1 ms which enables labeling times of 1 min and limits the labeling radius to 20 nm (Chu et al., 2017). After 1 min, the reaction is stopped with quenching buffer to prevent further biotinylation post-lysis. Cells may then be lysed before biotinylated proteins are isolated by streptavidin conjugated to beads and identified using MS-based workflows. An advantage of the short labeling timeframe is that APEX can be used to capture dynamic changes in PPIs. This may become advantageous for time course studies where protein interactions may dynamically change in response to various stimuli such as cell stress or drug treatments.

APEX can also be applied to analysis by electron microscopy (EM) (Martell et al., 2012; Lam et al., 2015), thereby enabling the interrogation of protein localization at high resolution. Here, fusion proteins are expressed in cells before cells are fixed and treated with diaminobenzidine (DAB) and H_2_O_2_ (Lam et al., 2015). APEX catalyzes the polymerization and local deposition of DAB in the vicinity of the fusion protein, enabling subsequent recruitment of electron dense osmium for EM applications (Lam et al., 2015).

By applying APEX for both proteomics and EM workflows, APEX can be used to characterize dynamic changes in the protein interactome of live cells whilst also accurately defining protein localization at high resolution. In recent years, variations of APEX have emerged. This includes APEX2 (Lam et al., 2015) which has improved catalytic efficiency and split APEX (sAPEX) which enables the interrogation of two known protein interaction partners or proteins known to be in close proximity (Han et al., 2018). Recently, the APEX technique has been applied to profile the components of stress granules, which are enriched in RNA-binding proteins including the ALS/FTD-linked proteins TDP-43 and FUS (Markmiller et al., 2018). Notably, these studies revealed previously unrecognized stress granules (SGs) proteins that also displayed alterations in induced pluripotent stem cell-derived motor neurons from ALS patients and for which modulation of expression was protective in *Drosophila* ALS models, suggesting disturbances in SGs may be related to disease pathogenesis (Markmiller et al., 2018).

### Future Applications of Proximity Labeling Techniques

Biotinylation by Antibody Recognition (BAR) is another proximity-labeling technique which has recently been established for investigating PPIs in fixed samples (Bar et al., 2018). Here, cultured cells or tissue are fixed and permeabilized before a primary antibody is used to target a protein of interest. A secondary antibody conjugated to horseradish peroxidase (HRP) is then used to recognize the primary antibody, and labeling of proximal proteins is achieved by HRP in the presence of H_2_O_2_ and biotin phenol. The conjugation of biotin enables the isolation of these proximal proteins. Although this method has not yet been applied for the study of neurodegenerative diseases, it is a promising technique for future analysis of proteins involved in ALS and FTD and may be particularly useful for analysis of detergent-insoluble protein aggregates and inclusions.

### Analysis of Protein Post-translational Modifications in ALS and FTD

Post-translational modifications provide the basis of biological diversity in the proteome by enabling the same protein to carry out diverse functions through characteristic variations of modifications and their temporal regulation. PTMs are small, covalent, amino acid modifications added onto a protein; they are highly dynamic and play key roles in selectively regulating protein function in cells, tissue, and biofluids, such as CSF (Guldbrandsen et al., 2014). PTMs regulate most aspects of intracellular function involving but not limited to, DNA repair, proliferation, subcellular localization, transport, and proteolytic cleavage of functional units or protein degradation. On a larger scale PTMs also modulate intercellular functions like cell signaling and adhesion (Minguez et al., 2012; Theillet et al., 2012; Olsen and Mann, 2013). Various types of PTMs exist (Prabakaran et al., 2012), with arguably the most studied in proteostasis and neurodegenerative diseases being: ubiquitination, phosphorylation, nitration, acetylation, oxidation, glycosylation, methylation, and sumoylation (Minguez et al., 2012).

Pathological phosphorylation of proteins such as tau, TDP-43, α-synuclein or FUS contribute to the formation of protein inclusions in neurodegenerative disease (Hasegawa et al., 2008; Despres et al., 2017; Monahan et al., 2017; Cykowski et al., 2018). For example, tauopathies caused by the phosphorylation of tau in FTD and AD are comprised of aggregated proteins in fibrillary tangles (Ferrer et al., 2002). While the underlying mechanisms of misfolded protein aggregation and neurotoxicity in neurodegeneration is not yet understood, PTMs such as ubiquitination are of interest given their regulatory role in protein degradation. For example, the temporal regulation of phosphorylation and ubiquitination in protein signaling cascades, and hyperubiquitination by mutant cyclin F cause defects to protein degradation pathways that are associated with ALS/FTD (Lee A. et al., 2017). Furthermore, other examples of aberrant PTMs include acetylation of glial fibrillary acidic protein in ALS (Liu et al., 2013), acetylated TDP-43 in ALS (Wang et al., 2017) and sialylation of amyloid precursor protein in AD (Nakagawa et al., 2006).

#### Detecting PTMs by Mass Spectrometry

Many PTMs can be mapped by tandem MS (MS/MS) (Dephoure et al., 2013), which offers an unbiased approach that can be verified by alternative biochemical methods such as immunoblotting and immunofluorescence microscopy. To date, the presence of PTMs such as ubiquitination, phosphorylation, acetylation, and nitration, and their stoichiometric analysis have been enabled by qualitative and quantitative proteomics, respectively, on a global proteomic scale in ALS-linked proteins (Sacksteder et al., 2006; Liu et al., 2013; Shi et al., 2013a; Lee A. et al., 2017; Wang et al., 2017). Despite the capabilities of MS to identify PTMs, they are often difficult to detect as they vary in physiochemical properties, can exist as transient modifications, be present in sub-stoichiometric concentrations and are sensitive to sample preparative steps that include high and low pH buffers, trypsinization, and de-salting steps (Wei and Li, 2008; Olsen and Mann, 2013). To circumvent many of the issues associated with analyzing PTMs such as ubiquitination and phosphorylation, enrichment strategies to isolate peptides containing these modifications together with quantitative proteomic approaches can provide a more in-depth analysis of the sub-proteome.

##### Ubiquitination

A popular enrichment strategy for identifying ubiquitination sites on peptides called diglycine (diGLY) enrichment exploits the cleavage sites (K-𝜀-GG) of ubiquitinated proteins following trypsin digestion (Kim et al., 2011). The covalent attachment of ubiquitin on lysine residues on proteins allows trypsin to cleave both the C-terminus of ubiquitin and the C-terminus of the lysine amino acid on the ubiquitinated protein exposing two glycine residues. Ubiquitin remnant motif (K-𝜀-GG) antibodies are used to enrich peptides containing the di-Gly-Gly motif, followed by elution and analysis by LC-MS/MS in which the presence of the GlyGly residue on lysine (+m/z 114.04) in the MS spectra is an indication of a ubiquitinated peptide (Peng et al., 2003; Xu et al., 2010; Fulzele and Bennett, 2018). However, ubiquitin-like proteins also exist that may be identified using this method such as SUMO (sumoylation), NEDD8 (neddylation) and interferon-stimulated gene 15 (ISG15; isg15ylation) also known as ubiquitin cross-reactive protein (UCRP) (Hemelaar et al., 2004). Quantitative assessment of the stoichiometry of site-specific ubiquitination can be achieved using the recently reported “isotopically balanced quantification of ubiquitination” (IBAQ-Ub) method which employs an amine-reactive chemical tag (AcGG-NHS) that is structurally homologous to a tryptically cleaved ubiquitinated peptide containing a GG remnant of ubiquitin on the modified lysine residue. These AcGG-NHS tagged peptides allows the generation of structurally identical peptides from ubiquitinated and unmodified lysine residues that can be further labeled using a secondary stable isotope (Li et al., 2019). Other areas which are generating interest are investigating the specific poly-ubiquitin linkages, in which isotope-labeling of the lysine residues in ubiquitin can be used, such as “UB-AQUA” and detected by high resolution MS via narrow window extracted ion chromatogram or by selected reaction monitoring (SRM-MS) using a triple quadrupole MS (Phu et al., 2011). Overall, these various methods enable stoichiometric analysis of the poly-ubiquitin modifications on target proteins, which could provide insights into the ubiquitin code underlying altered protein degradation in neurodegeneration.

##### Phosphorylation

Protein aggregates containing phosphorylated proteins is a key pathological feature in many neurodegenerative diseases, such as phospho-tau in Alzheimer’s disease and FTD (Sjogren et al., 2001), phospho-TDP-43 in ALS and FTD (Hasegawa et al., 2008) and phospho-alpha-synuclein in Parkinson’s disease (Wang et al., 2012). Phosphorylation of serine, threonine, and tyrosine residues are generally the most common sites in mammals, which are estimated to compromise of approximately 90, 10, and <1% of the total phosphoproteome, respectively (Nita-Lazar et al., 2008). The main challenges with characterizing phosphorylated proteins using MS is the direct detection of low stoichiometric concentrations of phosphopeptides resulting from trypsin digestion of complex samples. This is further complicated by ion suppression of phosphopeptides due to the negative charge and lability of the phosphate moiety. Therefore, many approaches have been developed to enrich phosphopeptides prior to MS-based analysis, including titanium dioxide (TiO_2_), immobilized metal affinity chromatography (IMAC), and hydrophilic interaction chromatography (HILIC), discussed elsewhere (Arrington et al., 2017). The detection of a phosphopeptide can be interpreted by the neutral loss of H_3_PO_4_ (-m/z 98) in the mass spectra. Many algorithms have been developed for interpreting MS/MS fragmentation spectra and phosphorylation site localization such as Mascot Delta Score (Savitski et al., 2011), PhosphoRS localization (Taus et al., 2011) and PTM score of MaxQuant (Cox and Mann, 2008). These algorithms match the spectra generated to a protein sequence database of choice (e.g., UniProtKB/Swiss-Prot) to assign the probabilities of potential phosphorylation sites.

Previously, sarkosyl-insoluble pathological TDP-43 from brains of two ALS patients were purified and subjected to LC-MS/MS analysis, identifying several novel phosphorylation sites, deamidation sites, and cleavage sites (Kametani et al., 2016). Seventeen phosphorylation sites were identified from both ALS patients which were predominately located in the Gly-rich C-terminal region on TDP-43, while most of the cleavage sites were located in N-terminal half, suggesting that these sites may be more accessible to proteolytic enzymes (Kametani et al., 2016). Immunoblot analysis using the phospho-specific TDP-43 S409/410 antibody, which recognizes the abnormal phosphorylation of Ser409 and 410, verified the presence of pathological phospho-forms of TDP-43 and additional fragments of 18∼24 kDa. These findings indicate that phosphorylation plays an important role in the mechanism of TDP-43 pathogenesis, and suggests that enrichment strategies to comprehensively characterize the phosphoproteome are highly relevant to understanding neurodegenerative diseases.

### Bioinformatics

Tandem mass spectra generated by MS are analyzed using specialized software and algorithms to identify and quantitate peptides and proteins using two main approaches: database search and *de novo* search. Database searching follows the “Exact Pattern-Matching rule,” which consists of only selecting spectra masses that exactly match a sequence in a multi-species- or species-specific database. The most frequently used programs with a database search implemented are SEQUEST (Eng et al., 1994), Mascot (Perkins et al., 1999), and X!Tandem (Craig and Beavis, 2004). These programs not only extract candidate proteins using a combination of the open access database and their own in-built database but also score them using algorithms based on the ion signals observed in the spectra. Some limitations of the database search method include that a large portion of acquired spectra will be rejected because of the “Exact Pattern-Matching rule” and not all organisms have a complete protein sequence reference.

*De novo* peptide sequencing generates a list of all the highest scored peptides from the MS/MS spectra and the mass values given without the need of a reference database. With high resolution mass spectrometers producing quality data, the performance of *de novo* sequencing has remarkably improved. However, a significant caveat pertains to resolving amino acids with or without modifications, due to either identical mass or near-identical masses. In addition, there is an inverse relationship between the accuracy of this method and peptide length (Muth and Renard, 2018). New strategies are being created in this space to overcome these limitations, such as *post novo*, which post-processes *de novo* sequence filter prediction (Miller et al., 2018).

Commonly used bioinformatics programs such as STRING (Szklarczyk et al., 2019), BioGrid (Oughtred et al., 2019), Ingenuity Pathway Analysis (IPA) (Yu J. et al., 2016), and DAVID (Huang da et al., 2009) are frequently used to interrogate the data obtained from large scale quantitative proteomic experiments to get a snapshot of the expressional changes that occur within a sub-proteome. Notably, depending on the biological question, these informatics tools can assist in quickly identifying networks, pathways and functions to facilitate further experiments and generate new hypotheses. For example, proteomics data generated from IP of TDP-43 from HEK293 lysates were analyzed by STRING, which demonstrated clear nuclear and cytoplasmic interaction networks (Freibaum et al., 2010). Assigning protein functions using bioinformatics software therefore provides a higher-level overview into changes and perturbations occurring during disease, for example allowing the identification of disease relevant biochemical pathways. A more detailed review of the bioinformatics tools used for proteomics is discussed elsewhere (Schmidt et al., 2014; Martens and Vizcaino, 2017).

### Flow Cytometric Purification of Inclusion Bodies Associated With ALS and FTD

Determination of the composition of insoluble inclusions formed in many neurodegenerative diseases is key to understanding pathogenesis. The use of proteomics will allow greater understanding of the global protein composition of these inclusions and allow us to identify proteins and cellular pathways that are either involved in the assembly or attempted disassembly of the inclusion, and distinguish these from proteins that were non-specifically sequestered. Comparing the protein composition of inclusions formed from a variety of pathogenic proteins (e.g., TDP-43- or SOD1-positive inclusions in ALS) could also help to establish common pathways involved in this process across different types of neurodegenerative diseases.

Fluorescence activated cell sorting (FACS) is one technique that can be used to purify insoluble protein or inclusions from cell culture or tissues. Regarding cell-based models of neurodegenerative disease-associated protein aggregation, gentle plasma membrane permeabilization in combination with FACS could be employed to purify inclusions for proteomic analyses. A method for this was recently described, whereby cells are permeabilized with 0.5% (v/v) TritonX-100 in PBS supplemented with protease/phosphatase inhibitors to quantify and characterize inclusions comprised of fluorescently tagged proteins of interest by flow cytometry (Whiten et al., 2016). This work also demonstrated that fluorescent aggregates and nuclei can be sorted by FACS (Whiten et al., 2016). Alternatively, cells can be treated with 0.03% (w/v) saponin to selectively permeabilize the plasma membrane, allowing soluble proteins to diffuse out of the cytoplasm, while retaining the nucleus and inclusions intact (Farrawell et al., 2018; Pokrishevsky et al., 2018). These treated cells can be washed to remove the soluble proteins that have diffused out of the cells and subsequently purify cells with inclusions by FACS. It follows that inclusions comprised of fluorescently tagged proteins, or fluorescent antibody-labeled inclusions can be purified by FACS from cells and tissues and subjected to proteomic analysis to determine their composition.

Purification of inclusions by FACS for proteomic analysis has previously been adopted to investigate the protein composition of Huntington’s disease-associated polyglutamine inclusions (Mitsui et al., 2002, 2006). Using this strategy, heat shock protein 84 and elongation factor 1α were identified as novel inclusion-interacting proteins and their over-expression in cell-based assays demonstrated cytoprotective activities (Mitsui et al., 2002, 2006). Future experiments could employ this strategy to purify insoluble protein and inclusions for proteomic analysis from ALS/FTD patient samples, rodent tissues, or cell-based models of ALS/FTLD.

## Biomarker Studies of Als and Ftd

As high-throughput technologies have advanced, many proteomic-based biomarker studies have presented an abundance of potential candidates; however, it is often not feasible to properly validate each individual protein considering the heterogeneity amongst different patient cohorts, which can hinder the level of accuracy and reproducibility. Selection of which candidates to validate is not clearly defined, uniform, or standardized. This selection can be made based on a combination of statistics, current literature as well as assessed potential based on PPIs, both predicted and real.

One of the major issues that arises as a result of subjective validation is the variation of proteins identified across parallel studies, rarely reinforcing the previously presented data. For example, measurements of HeLa cells prepared identically and analyzed across different laboratories using the same instrumentation revealed a high level of biological heterogeneity on both transcriptomic and proteomic levels (Liu et al., 2019). This occurs for a number of reasons, including the sample composition and sample cohorts, environmental conditions affecting instrument parameters, as well as technical variation due to extraction/digestion procedures (Piehowski et al., 2013). Alternatively, the same protein could be reported with differing results across samples, such as decreased levels of galectin-3 in the plasma of SOD1 mice (Zubiri et al., 2018) and increased levels in human CSF (Zhou et al., 2010). These results are not necessarily conflicting but highlight the importance of validation across multiple large cohorts for biological relevance.

In recent years, an increased number of small and large-scale biomarker studies have been performed on human ALS samples by LFQ, as recently reviewed (Barschke et al., 2017). CSF from ALS, primary lateral sclerosis (PLS), cross-sectional healthy and disease controls (Parkinsons’ disease and ALS mimic disorders) were analyzed via LFQ for novel biomarker discovery (Thompson et al., 2018). Elevation in levels of chitotriosidase, chitinase-3-like protein 1 and chitinase-3-like protein 2 in a cohort of 43 patients with ALS has been revealed, indicating neuroinflammation due to increased microglial activation (Thompson et al., 2018). No direct validation was performed to confirm the proteomic data, although reference to previous ELISA studies of these chitinase proteins in ALS patient samples (likely including some from the same cohort), suggested a similar trend (Varghese et al., 2013).

Biofluid sample usage is prominent due to the need for a directly translatable and easily retrievable marker for rapid and accurate diagnosis. Alongside disease diagnosis, the ability to distinguish between subtypes of a disease would prove instrumental in developing personalized treatments. Whilst many studies have identified altered proteins across cohorts of patients, others focus on teasing apart unclear differences such as those found in subtypes of FTD (Teunissen et al., 2016). Recently, a CSF-wide proteomic analysis was performed to highlight the usefulness of biofluid MS for biomarker elucidation, identifying relevant disease-related proteins such as tau (Macron et al., 2018). In the context of biomarker discovery, newer labeled methods are being utilized to narrow down on protein hits of interest due to the greater quantitation accuracy compared to label-free methods (Megger et al., 2014). A summary of ALS and FTD biomarker elucidation studies in the last decade that have used both label-free and labeled methods is presented in Table 6. Ultimately, it is crucial to have a clear biological question to pursue for selection of sample, technique and subsequent validation approaches, although unbiased screening of potential disease-related biofluid alterations may help identify previously unrecognized research directions.

**Table 6:** Biomarker discovery studies using proteomics for ALS and FTD.

Technique	Sample	Summary	Remarks	References
iTRAQ	ALS CSF	↓ IGF-2, ↑ GRIA4 (levels correlate with ALS severity)	Potential biomarker for disease severity (gender differences)	Chen et al., 2016
iTRAQ	sALS CSF	31 proteins ↑ in ALS CSF, ↑ CHIT1 10x	CHIT1 expression = ↑ microglial activity (potential CSF biomarker)	Varghese et al., 2013
Label free	TDP-43/Tau FTD CSF and blood	56 proteins ↑/↓ in FTD subtypes (notably CHI3L-1 and CAT)	Requires validation in FTD pathology cohorts	Teunissen et al., 2016
Label-free	ALS CSF and plasma	27 proteins ↑/↓ in ALS CSF, 20 proteins ↑/↓ in ALS plasma (Validated CHI3L-1 and ACT)	New and previously identified potential biomarkers	Bereman et al., 2018
Label-free	ALS CSF	↑ CHIT1, CHI3L-1, and CHI3L-2	Neuroinflammatory mechanisms implicated through microglial activation	Thompson et al., 2018
Label-free	sALS plasma	Serum proteome characterization	Lipid homeostasis proteins implicated	De Benedetti et al., 2017
Label-free	sALS CSF	WDR63, APLP2, SPARCL1, and CADM3 = potential biomarkers	Panel of CSF proteins for biomarkers	Collins et al., 2015
Label-free	sALS and other neural diseases	↑ MyBP-H potential ALS biomarker compared to other motor neuropathies	↑ MyBP-H = attempted regeneration	Conti et al., 2014
Label-free	SOD1^G93A^ mouse spinal cord, ALS spinal cord/CSF	14 proteins ↑/↓, ↑ Gal-3 in ALS	Comparative study of mouse and human ALS	Zhou et al., 2010
Label-free (MRM)	ALS and FTD blood, CSF and brain	TDP-43 in CSF appears to originate from the blood	TDP-43 in blood and CSF not a viable biomarker	Feneberg et al., 2014
Label-free (SRM)	SOD1^G93A^ mouse muscles	Shift in fiber-type composition of hindlimb muscles	Diagnostic-prognostic tool for neuromuscular diseases via myosin heavy chain isoform?	Peggion et al., 2017
MALDI	ALS blood	↑/↓blood coagulation proteins	SVM-based model with >97% recognition capability and >90% specificity and sensitivity	Conraux et al., 2013
MALDI	SOD1^G93A^ mouse muscle	↑/↓ muscle albumin, complex I, complex II, citrate synthase, FAS, PI3K, PGC1α, SEMA-3A semaphorin-3A, ROCK1	Potential dysmetabolism molecular signatures in ALS	Capitanio et al., 2012
MALDI	sALS and SOD1^G93A^ rat blood	↑/↓ ER stress, nitrative stress, redox, RNA metabolism proteins	Related to pathogenic mechanisms in ALS	Nardo et al., 2011
MALDI	sALS and SOD1^G93A^ rat blood	↑ protein nitration early in disease	Nitrated proteins = presymptomatic biomarker for ALS	Nardo et al., 2009
MALDI and label-free	sALS and fALS (nonSOD1) plasma	GC2 allele of DBP = potential risk factor for fALS	Small cohort, requires validation	Palma et al., 2008
SILAC	ALS fibroblasts	33 proteins ↑/↓ (↓ ApoB48 and HSP20, ↑ FIBL-1)	Potential biomarkers/therapeutic targets	Narayan et al., 2016
Stable isotope dimethyl-labeling	ALS tibialis anterior muscle	↑/↓ KBTBD10, MYL3, AGL, and VCP	Potential muscle biopsy biomarkers and therapeutic targets	Elf et al., 2014
SWATH	ALS plasma	↑/↓ GSN, APOJ, CDL5, and FCN3	↓ coagulation pathway proteins and ↑ complement pathway proteins	Xu et al., 2018
TMT	SOD1^G93A^ mouse and sALS plasma	↑/↓ApoE in human and Gal-3, CD61 and TGF-β1 in SOD1^G93A^ mice	Altered immunosenescence and metabolic markers	Zubiri et al., 2018

*↑, denotes increase; ↓, denotes decrease; ACT, alpha-1-antichymotrypsin; AGL, glycogen debranching enzyme; APLP2, amyloid-like protein 1; ApoE, apolipoprotein E; CADM3, cell adhesion molecule 3; CAT, catalase; CD5L, CD5 antigen-like; CD61, integrin beta 3; CHI3L-1, chitinase 3-like protein 1; CHI3L-2, chitinase 3-like protein 2; CHIT1, chitotriosidase-1; CSF, cerebrospinal fluid; DBP, vitamin D-binding protein; fALS, familial amyotrophic lateral sclerosis; FAS, tumor necrosis factor receptor superfamily member 6; FCN3, ficolin 3; FIBL-1, fibulin-1; FTD, frontotemporal dementia; Gal-3, galectin-3; GRIA4, glutamate receptor 4; GSN, gelosin, APOJ, clusterin; IGF-2, insulin growth factor II; iTRAQ, isobaric tags for relative and absolute quantitation; KBTBD10, BTB and kelch domain-containing protein 10; MALDI, matrix-assisted laser desorption ionization; MRM, multiplex reaction monitoring; MYL3, myosin light chain 3; PGC1α, peroxisome proliferator-activated receptor gamma coactivator 1-alpha; PI3K, phosphoinositide 3-kinase; ROCK1, rho-associated protein kinase 1; sALS, sporadic amyotrophic lateral sclerosis; SEMA-3A, semaphorin-3A; SILAC, stable isotope labeling by amino acids in cell culture; SOD1, superoxide dismutase 1; SPARCL1, SPARC-like protein 1; SRM, selected reaction monitoring; SWATH, sequential window acquisition of all theoretical mass spectra; TDP-43, TAR DNA binding protein of 43 kDa; TGF-β1, transforming growth factor beta-1; TMT, tandem mass tag; VCP, transitional ER ATPase; and WDR63, WD repeat-containing protein 63.*

## Future Directions and Conclusion

The technologies presented in this review have demonstrated their utility when applied to neurodegenerative diseases such as ALS and FTD. However, there needs to be a focus on validating the research laterally, solidifying findings to progress with ensuing biological questions. Incorporation of parallel approaches such as transcriptomics using RNASeq alongside results from proteomics can reinforce potential hits of interest. Studies have successfully combined transcriptomic and proteomics findings to provide mechanistic insights, such as the finding of lysosomal changes in Grn-/- mice that are modulated by the FTD-risk protein TMEM106B (Klein et al., 2017). Additionally, recent advances in this area have included correlation of transcriptomic findings with underlying TDP-43 pathology burden in a study of ALS spinal cord laser-capture dissected motor neurons (Krach et al., 2018). This was one of the first studies to utilize overlapping distinct datasets to stratify patient cohorts to identify the more relevant and important proteins involved in disease. Previously, careful patient sub-type selection has led to the identification of genetic contributors to disease, such as the finding that TMEM106b genotype is a risk modifier of FTD by GWAS of a highly specific and well-defined subgroup of FTLD samples with confirmed TDP-43 histopathology (Van Deerlin et al., 2010). These findings suggest that planning sample selection to allow multiple comparisons of disease subtypes, alongside the combined use of new protein- and RNA-profiling technologies, will lead to further advances in the field of ALS and FTD research.

Each technique discussed in this review has advantages and limitations, described above and in additional detail elsewhere (Johnson et al., 2005; Parker et al., 2009; Neilson et al., 2011; Megger et al., 2014; Arrington et al., 2017; Lindemann et al., 2017; Martens and Vizcaino, 2017; Nadler et al., 2017; Tholey and Becker, 2017). Importantly, issues of reproducibility present in less frequently used and outdated techniques such as surface-enhanced laser desorption/ionization (SELDI) have been addressed by the introduction of newer technologies. Whole proteome screening at high resolutions has also replaced the need for “precision” techniques such as two-dimensional polyacrylamide gel electrophoresis (2D-PAGE) coupled to MS. The labeling of proximal proteins to investigate PPIs using techniques such as BAR will allow for more complex investigation that is not currently provided by standard affinity chromatography methods. The implementation of novel and high-throughput techniques in ALS/FTD can be used to overcome current issues in variability between studies as well as the low correlation of findings between differing studies. Furthermore, the application of different techniques is context-dependent, and choice of technology must take into account both biological and technical considerations. In addition, it is crucial that both experimental approaches and data interpretation are considered in study design.

In this review we have identified proteomics papers relevant to ALS/FTD and presented them alongside discussion of current technologies. The consistency amongst proteins identified strictly depend on the disease type/model used, however, in ALS research, TDP-43 as well as hnRNPs appear to reoccur (Ling et al., 2013). An assessment as to whether these commonalities are consistent has not been carried out as these would have to account for differences in stress (oxidative/heat) as well as cell type (neuron/cell-line/species).

In the context of ALS and FTD, greater focus should be given to comparisons between new and existing proteomics datasets as the growing number of studies are released using a variety of available techniques. Further comparisons between ALS and FTD datasets and those obtained from other neurodegenerative diseases should also be performed, since there are likely to be overlap in mechanisms which may be broadly involved in neurodegeneration. This may also provide information on how disease-specific alterations arise and why certain cell populations or CNS regions are susceptible to different neurodegenerative disease processes. For example, recent studies have applied TMT to identify changes in various Alzheimer’s disease mice, identifying hundreds of differentially expressed proteins (Kim et al., 2018). Previously, these comparisons have also been used for biomarker elucidation, attempting to highlight differences between similar neurodegenerative diseases such as FTD and Alzheimer’s disease (Teunissen et al., 2016).

Finally, it will also be informative to use these proteomic techniques to more closely investigate biochemical changes longitudinally in disease, particularly in studies of model systems from which brain and spinal cord samples can be collected at pre-defined time points. Strategies such as this are most likely to reveal the earliest pathogenic changes in disease, which may be most amenable to therapeutic intervention, but which may not be identified by studies at later disease time points or by using end-stage human tissue samples alone. The use of comparative studies and recent advances in new technologies and labeling techniques offers great hope for future understanding of ALS and FTD, and development of clinical tests and therapeutics based on these findings.

## Author Contributions

TH, AW, and AL conceived of the manuscript and drafted the tables. All authors were responsible for drafting, writing and editing, and read and approved the final manuscript.

## Conflict of Interest Statement

The authors declare that the research was conducted in the absence of any commercial or financial relationships that could be construed as a potential conflict of interest.
